# Poly[[diaqua­bis­(μ_3_-isonicotinato-κ^3^ *N*:*O*:*O*′)bis­(μ_2_-isonicotinato-κ^2^ *N*:*O*)gadolinium(III)disiliver(I)] nitrate monohydrate]

**DOI:** 10.1107/S1600536810035634

**Published:** 2010-09-11

**Authors:** Le-Qing Fan, Ji-Huai Wu

**Affiliations:** a Institute of Materials Physical Chemistry, and The Key Laboratory for Functional Materials of Fujian Higher Education, Huaqiao University, Quanzhou, Fujian 362021, People’s Republic of China

## Abstract

In the title compound, {[Ag_2_Gd(C_6_H_4_NO_2_)_4_(H_2_O)_2_]NO_3_·H_2_O}_*n*_, the Gd^III^ ion is coordinated by eight O atoms from six isonicotinate ligands and two water mol­ecules in a distorted square anti­prismatic geometry. Two Ag^I^ ions are each bonded to two N atoms from two isonicotinate ligands in a linear or bow-like fashion [N—Ag—N angles = 178.6 (2) and 147.1 (2)°]. These metal ions are connected by the isonicotin­ate ligands into a layer parallel to (010). O—H⋯O hydrogen bonds donated by the coordinated and uncoordinated water mol­ecules and intra­layer π–π stacking inter­actions between the pyridine rings [centroid–centroid distances = 3.551 (4) and 3.555 (4) Å] are observed. The layers inter­act with each other by inter­layer Ag⋯O(aqua) contacts [2.731 (4) Å] and π–π stacking inter­actions between the pyridine rings [centroid–centroid distances = 3.466 (3) and 3.516 (3) Å], resulting in the formation of a three-dimensional supra­molecular structure.

## Related literature

For general background to the structures and properties of lanthanide–transition metal coordination polymers, see: Cheng *et al.* (2007 ▶, 2008 ▶); Fan & Wu (2010 ▶); Fang *et al.* (2009 ▶); Luo *et al.* (2007 ▶).
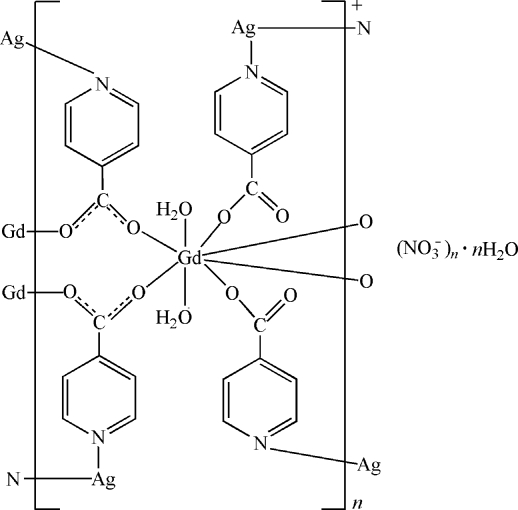


## Experimental

### 

#### Crystal data

[Ag_2_Gd(C_6_H_4_NO_2_)_4_(H_2_O)_2_]NO_3_·H_2_O
*M* *_r_* = 977.46
Monoclinic, 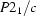

*a* = 16.889 (8) Å
*b* = 24.744 (11) Å
*c* = 6.750 (3) Å
β = 96.240 (9)°
*V* = 2804 (2) Å^3^
*Z* = 4
Mo *K*α radiation
μ = 3.80 mm^−1^
*T* = 293 K
0.30 × 0.12 × 0.08 mm

#### Data collection

Rigaku Mercury CCD diffractometer
Absorption correction: multi-scan (*CrystalClear*; Rigaku, 2007 ▶) *T*_min_ = 0.703, *T*_max_ = 1.000
16445 measured reflections
4859 independent reflections
4370 reflections with *I* > 2σ(*I*)
*R*_int_ = 0.032

#### Refinement

*R*[*F* ^2^ > 2σ(*F* ^2^)] = 0.034
*wR*(*F* ^2^) = 0.129
*S* = 1.08
4859 reflections
416 parameters
H-atom parameters constrained
Δρ_max_ = 1.38 e Å^−3^
Δρ_min_ = −1.27 e Å^−3^

### 

Data collection: *CrystalClear* (Rigaku, 2007 ▶); cell refinement: *CrystalClear*; data reduction: *CrystalClear*; program(s) used to solve structure: *SHELXS97* (Sheldrick, 2008 ▶); program(s) used to refine structure: *SHELXL97* (Sheldrick, 2008 ▶); molecular graphics: *SHELXTL* (Sheldrick, 2008 ▶) and *DIAMOND* (Brandenburg, 1999 ▶); software used to prepare material for publication: *SHELXTL* and *PLATON* (Spek, 2009 ▶).

## Supplementary Material

Crystal structure: contains datablocks global, I. DOI: 10.1107/S1600536810035634/hy2346sup1.cif

Structure factors: contains datablocks I. DOI: 10.1107/S1600536810035634/hy2346Isup2.hkl

Additional supplementary materials:  crystallographic information; 3D view; checkCIF report

## Figures and Tables

**Table 1:** Selected bond lengths (Å)

Gd1—O1	2.357 (4)
Gd1—O2^i^	2.465 (4)
Gd1—O3	2.383 (4)
Gd1—O5	2.393 (4)
Gd1—O7	2.399 (4)
Gd1—O8^i^	2.386 (4)
Gd1—O9	2.454 (4)
Gd1—O10	2.533 (4)
Ag1—N1	2.144 (5)
Ag1—N4^ii^	2.147 (5)
Ag2—N2^iii^	2.189 (5)
Ag2—N3	2.199 (5)

Symmetry codes: (i) 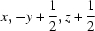; (ii) 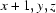; (iii) 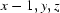.

**Table 2:** Hydrogen-bond geometry (Å, °)

*D*—H⋯*A*	*D*—H	H⋯*A*	*D*⋯*A*	*D*—H⋯*A*
O9—H9*A*⋯O6	0.85	2.13	2.771 (7)	132
O9—H9*B*⋯O2^iv^	0.85	2.03	2.811 (6)	153
O10—H10*A*⋯O6^v^	0.85	2.12	2.954 (6)	165
O10—H10*C*⋯O4	0.85	1.84	2.662 (6)	162
O14—H14*B*⋯O12^vi^	0.85	2.27	2.960 (9)	139
O14—H14*C*⋯O13^vii^	0.85	2.01	2.779 (10)	151

Symmetry codes: (iv) 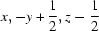; (v) 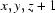; (vi) 
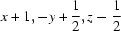
; (vii) 
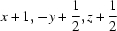
.

**Fig. 1.**
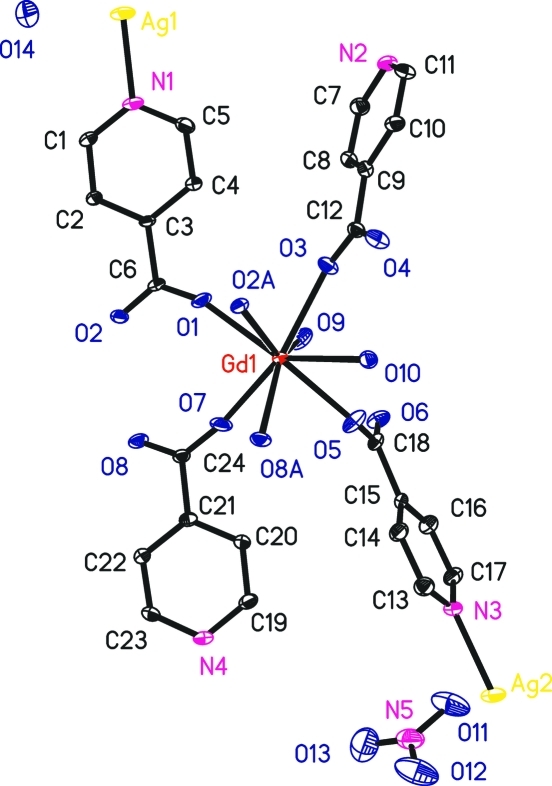
The asymmetric unit of the title compound. Displacement ellipsoids are drawn at the 30% probability level. Additional symmetry related atoms are included to complete the coordination geomtry around the Gd atom. [Symmetry code: (A) x, 1/2-y, 1/2+z.]

**Fig. 2.**
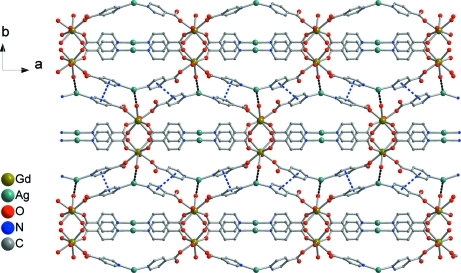
The three-dimensional supramolecular structure viewed along the c axis, formed through weak interlayer Ag···O contacts and π–π stacking interactions (dashed lines).

## supplementary crystallographic information

### Comment

In recent years, investigations on the design and synthesis of lanthanide-transition metal coordination polymers have attracted great interest not only for their fascinating structural topologies but also for their potential applications in magnetism, luminescence materials, molecular adsorption, and bimetallic catalysis (Cheng *et al.*, 2007, 2008; Fan & Wu, 2010; Fang *et al.*, 2009; Luo *et al.*, 2007). Isonicotinic acid, which acts as a multidentate ligand possessing N and O donor atoms, is utilized to construct lanthanide-transition metal coordination polymers *via* the carboxylate group coordinating to lanthanide ions and N atom bonding to transition metal ions, such as Ag^I^ or Cu^I^ ions. We report herein the crystal structure of the title 4d-4f compound by the reaction of Gd_2_O_3_, isonicotinic acid and AgNO_3_ under hydrothermal conditions.

As shown in Fig. 1, the asymmetric unit of the title compound contains one Gd^III^ ion, two Ag^I^ ions, four isonicotinate ligands, two coordinated water molecules, one nitrate ion, and one uncoordinated water molecule. The Gd^III^ ion is coordinated by eight O atoms from six isonicotinate ligands and two water molecules in a distorted square antiprismatic geometry, with the Gd—O bond lengths and O—Gd—O bond angles being from 2.356 (4) to 2.534 (4) Å and 71.22 (13) to 145.29 (14)°, respectively (Table 1). Each Ag^I^ ion is bonded to two N atoms from two different isonicotinate ligands in a linear or bow-like fashion, with the Ag—N bond lengths of 2.145 (5)–2.201 (5) Å and N—Ag—N bond angles of 178.5 (2)° and 147.30 (19) (Table 1). Adjacent Gd centers are connected by two carboxylate groups from two different isonicotinate ligands, forming one-dimensional chains, which are further linked by Ag^I^ ions to construct two-dimensional layers. The layers are stabilized by O—H···O hydrogen bonds involving the coordinated and uncoordinated water molecules (Table 2) and intralayer π–π stacking interactions between the pyridine rings, with centroid–centroid distances of 3.551 (4) and 3.555 (4) Å (Spek, 2009. The layers interact each other by interlayer Ag2···O10(aqua) contacts [2.731 (4) Å] and π–π stacking interactions between the pyridine rings from two neighboring layers, with centroid–centroid distances of 3.466 (3) and 3.516 (3) Å, which result in the formation of a three-dimensional supramolecular structure (Fig. 2).

### Experimental

A mixture of Gd_2_O_3_ (0.181 g, 0.5 mmol), isonicotinic acid (0.123 g, 1 mmol), AgNO_3_ (0.170 g, 1 mmol) and H_2_O (10 ml) was placed in a 23 ml Teflon-lined reactor, which was heated to 443 K for 7 d and then cooled to room temperature at a rate of 0.2 K h^-1^. The colorless crystals obtained were washed with water and dried in air (yield 32% based on Gd).

### Refinement

H atoms were positioned geometrically and refined as riding atoms, with C—H = 0.93 and O—H = 0.85 Å and *U*_iso_(H) = 1.2*U*_eq_(C, O). The highest residual electron density was found 1.18 Å from O10 and the deepest hole 0.81 Å from Gd1.

### Crystal data

**Table:**

[Ag_2_Gd(C_6_H_4_NO_2_)_4_(H_2_O)_2_]NO_3_·H_2_O	*F*(000) = 1884
*M**_r_* = 977.46	*D*_x_ = 2.315 Mg m^−^^3^
Monoclinic, *P*2_1_/*c*	Mo *K*α radiation, λ = 0.71073 Å
Hall symbol: -P 2ybc	Cell parameters from 6663 reflections
*a* = 16.889 (8) Å	θ = 3.0–27.5°
*b* = 24.744 (11) Å	µ = 3.80 mm^−^^1^
*c* = 6.750 (3) Å	*T* = 293 K
β = 96.240 (9)°	Block, colorless
*V* = 2804 (2) Å^3^	0.30 × 0.12 × 0.08 mm
*Z* = 4	

### Data collection

**Table:**

Rigaku Mercury CCD diffractometer	4859 independent reflections
Radiation source: fine-focus sealed tube	4370 reflections with *I* > 2σ(*I*)
graphite	*R*_int_ = 0.032
ω scans	θ_max_ = 25.0°, θ_min_ = 2.6°
Absorption correction: multi-scan (*CrystalClear*; Rigaku, 2007)	*h* = −20→19
*T*_min_ = 0.703, *T*_max_ = 1.000	*k* = −28→29
16445 measured reflections	*l* = −8→8

### Refinement

**Table:**

Refinement on *F*^2^	Primary atom site location: structure-invariant direct methods
Least-squares matrix: full	Secondary atom site location: difference Fourier map
*R*[*F*^2^ > 2σ(*F*^2^)] = 0.034	Hydrogen site location: inferred from neighbouring sites
*wR*(*F*^2^) = 0.129	H-atom parameters constrained
*S* = 1.08	*w* = 1/[σ^2^(*F*_o_^2^) + (0.0925*P*)^2^ + 0.0116*P*] where *P* = (*F*_o_^2^ + 2*F*_c_^2^)/3
4859 reflections	(Δ/σ)_max_ = 0.002
416 parameters	Δρ_max_ = 1.38 e Å^−^^3^
0 restraints	Δρ_min_ = −1.27 e Å^−^^3^

### Fractional atomic coordinates and isotropic or equivalent isotropic displacement parameters (Å^2^)

**Table:**

	*x*	*y*	*z*	*U*_iso_*/*U*_eq_	
Gd1	0.769985 (15)	0.162835 (10)	0.61682 (4)	0.01965 (14)	
Ag1	1.27129 (3)	0.27098 (2)	0.49007 (9)	0.04268 (18)	
Ag2	0.26502 (3)	0.00962 (2)	0.40147 (9)	0.04637 (19)	
O1	0.8613 (2)	0.23037 (17)	0.5464 (8)	0.0399 (11)	
O2	0.8530 (2)	0.31392 (15)	0.4284 (6)	0.0255 (8)	
O3	0.8850 (2)	0.10782 (16)	0.6024 (6)	0.0309 (9)	
O4	0.9135 (3)	0.05792 (18)	0.8754 (6)	0.0342 (10)	
O5	0.6664 (3)	0.1044 (2)	0.4728 (6)	0.0412 (12)	
O6	0.6514 (3)	0.08461 (19)	0.1515 (7)	0.0380 (11)	
O7	0.6853 (2)	0.22994 (18)	0.4473 (7)	0.0394 (11)	
O8	0.6733 (2)	0.31472 (17)	0.3362 (6)	0.0305 (9)	
O9	0.7837 (3)	0.1483 (2)	0.2626 (6)	0.0419 (12)	
H9A	0.7661	0.1161	0.2502	0.050*	
H9B	0.8157	0.1639	0.1924	0.050*	
O10	0.7581 (2)	0.08017 (15)	0.8329 (6)	0.0293 (9)	
H10C	0.8059	0.0717	0.8722	0.035*	
H10A	0.7353	0.0817	0.9390	0.035*	
O11	0.2993 (4)	0.0486 (3)	0.8246 (9)	0.076 (2)	
O12	0.2422 (4)	0.0839 (3)	1.0578 (9)	0.084 (2)	
O13	0.2575 (5)	0.1295 (3)	0.7976 (11)	0.083 (2)	
O14	1.2728 (4)	0.3429 (2)	0.9058 (12)	0.0660 (18)	
H14C	1.2816	0.3432	1.0322	0.08 (4)*	
H14B	1.2406	0.3619	0.8292	0.095*	
N1	1.1448 (3)	0.2720 (2)	0.4982 (7)	0.0295 (11)	
N2	1.1488 (3)	0.0291 (2)	0.4987 (8)	0.0319 (12)	
N3	0.3875 (3)	0.0365 (2)	0.3745 (8)	0.0317 (11)	
N4	0.3979 (3)	0.2720 (2)	0.4807 (8)	0.0326 (12)	
N5	0.2680 (4)	0.0875 (3)	0.8983 (11)	0.0524 (17)	
C1	1.1038 (3)	0.3187 (3)	0.4945 (9)	0.0300 (13)	
H1A	1.1321	0.3510	0.4970	0.036*	
C2	1.0215 (3)	0.3210 (2)	0.4872 (9)	0.0254 (12)	
H2A	0.9952	0.3541	0.4804	0.030*	
C3	0.9793 (3)	0.2732 (2)	0.4900 (8)	0.0192 (11)	
C4	1.0219 (3)	0.2248 (2)	0.4990 (9)	0.0244 (12)	
H4A	0.9951	0.1920	0.5020	0.029*	
C5	1.1036 (4)	0.2255 (3)	0.5035 (9)	0.0306 (13)	
H5A	1.1312	0.1929	0.5103	0.037*	
C6	0.8906 (3)	0.2727 (2)	0.4838 (7)	0.0202 (11)	
C7	1.0969 (4)	0.0607 (2)	0.3909 (10)	0.0319 (13)	
H7A	1.1093	0.0724	0.2670	0.038*	
C8	1.0253 (4)	0.0770 (2)	0.4541 (9)	0.0286 (13)	
H8A	0.9897	0.0980	0.3723	0.034*	
C9	1.0080 (3)	0.0614 (2)	0.6418 (8)	0.0209 (11)	
C10	1.0615 (3)	0.0291 (2)	0.7561 (9)	0.0299 (13)	
H10B	1.0514	0.0180	0.8826	0.036*	
C11	1.1301 (4)	0.0136 (2)	0.6792 (10)	0.0325 (14)	
H11A	1.1654	−0.0089	0.7558	0.039*	
C12	0.9283 (3)	0.0766 (2)	0.7152 (8)	0.0229 (12)	
C13	0.4339 (4)	0.0597 (3)	0.5286 (9)	0.0327 (14)	
H13A	0.4127	0.0643	0.6490	0.039*	
C14	0.5102 (4)	0.0765 (2)	0.5146 (10)	0.0326 (14)	
H14A	0.5406	0.0910	0.6250	0.039*	
C15	0.5418 (3)	0.0717 (2)	0.3342 (8)	0.0219 (11)	
C16	0.4947 (4)	0.0496 (2)	0.1764 (8)	0.0294 (13)	
H16A	0.5139	0.0462	0.0530	0.035*	
C17	0.4192 (4)	0.0327 (3)	0.2018 (10)	0.0328 (14)	
H17A	0.3883	0.0178	0.0930	0.039*	
C18	0.6282 (3)	0.0883 (2)	0.3187 (8)	0.0231 (12)	
C19	0.4396 (3)	0.2255 (2)	0.4717 (9)	0.0288 (13)	
H19A	0.4125	0.1928	0.4741	0.035*	
C20	0.5204 (3)	0.2245 (2)	0.4593 (8)	0.0234 (12)	
H20A	0.5476	0.1918	0.4597	0.028*	
C21	0.4397 (4)	0.3187 (3)	0.4771 (9)	0.0323 (14)	
H21A	0.4130	0.3511	0.4912	0.039*	
C22	0.5186 (3)	0.3206 (2)	0.4539 (9)	0.0260 (12)	
H22A	0.5441	0.3536	0.4434	0.031*	
C23	0.5604 (3)	0.2726 (2)	0.4462 (7)	0.0202 (11)	
C24	0.6471 (3)	0.2728 (2)	0.4095 (8)	0.0232 (12)	

### Atomic displacement parameters (Å^2^)

**Table:**

	*U*^11^	*U*^22^	*U*^33^	*U*^12^	*U*^13^	*U*^23^
Gd1	0.0133 (2)	0.0206 (2)	0.0256 (2)	−0.00073 (9)	0.00462 (13)	0.00042 (9)
Ag1	0.0119 (3)	0.0577 (4)	0.0593 (4)	−0.0001 (2)	0.0077 (2)	0.0006 (3)
Ag2	0.0197 (3)	0.0572 (4)	0.0642 (4)	0.0012 (2)	0.0137 (2)	0.0041 (3)
O1	0.016 (2)	0.032 (2)	0.071 (3)	−0.0077 (18)	0.004 (2)	0.019 (2)
O2	0.0152 (19)	0.028 (2)	0.033 (2)	0.0050 (17)	−0.0015 (16)	0.0059 (17)
O3	0.027 (2)	0.030 (2)	0.037 (2)	0.0107 (18)	0.0090 (19)	0.0024 (18)
O4	0.033 (2)	0.042 (3)	0.029 (2)	0.016 (2)	0.0073 (19)	0.0101 (19)
O5	0.036 (3)	0.056 (3)	0.030 (2)	−0.026 (2)	−0.002 (2)	−0.001 (2)
O6	0.029 (2)	0.054 (3)	0.033 (2)	−0.010 (2)	0.0116 (19)	−0.005 (2)
O7	0.016 (2)	0.041 (3)	0.061 (3)	0.0114 (19)	0.004 (2)	0.015 (2)
O8	0.019 (2)	0.035 (2)	0.039 (2)	−0.0045 (18)	0.0097 (18)	0.0090 (19)
O9	0.041 (3)	0.063 (3)	0.023 (2)	−0.022 (2)	0.0083 (19)	−0.002 (2)
O10	0.026 (2)	0.025 (2)	0.038 (2)	0.0025 (18)	0.0141 (18)	0.0036 (17)
O11	0.051 (4)	0.112 (6)	0.063 (4)	0.029 (4)	0.002 (3)	−0.033 (4)
O12	0.065 (4)	0.143 (7)	0.042 (3)	0.047 (4)	−0.002 (3)	−0.002 (3)
O13	0.102 (6)	0.068 (5)	0.081 (4)	−0.019 (4)	0.020 (4)	−0.007 (4)
O14	0.062 (4)	0.038 (3)	0.098 (6)	−0.002 (3)	0.007 (4)	−0.001 (3)
N1	0.015 (2)	0.042 (3)	0.032 (3)	−0.001 (2)	0.005 (2)	0.000 (2)
N2	0.018 (3)	0.034 (3)	0.045 (3)	−0.002 (2)	0.009 (2)	−0.006 (2)
N3	0.016 (2)	0.032 (3)	0.047 (3)	0.002 (2)	0.003 (2)	0.002 (2)
N4	0.018 (3)	0.036 (3)	0.045 (3)	0.001 (2)	0.012 (2)	−0.003 (2)
N5	0.030 (3)	0.063 (4)	0.064 (5)	0.002 (3)	0.006 (3)	−0.018 (4)
C1	0.016 (3)	0.030 (3)	0.044 (3)	−0.008 (3)	0.006 (3)	0.000 (3)
C2	0.015 (3)	0.023 (3)	0.039 (3)	−0.001 (2)	0.008 (2)	0.005 (2)
C3	0.014 (3)	0.022 (3)	0.022 (3)	−0.001 (2)	0.002 (2)	0.002 (2)
C4	0.016 (3)	0.023 (3)	0.034 (3)	0.000 (2)	0.003 (2)	0.001 (2)
C5	0.024 (3)	0.031 (3)	0.037 (3)	0.007 (3)	0.005 (3)	0.003 (3)
C6	0.016 (3)	0.027 (3)	0.018 (2)	−0.004 (2)	0.003 (2)	−0.004 (2)
C7	0.028 (3)	0.031 (3)	0.039 (3)	0.002 (3)	0.012 (3)	0.002 (3)
C8	0.023 (3)	0.024 (3)	0.040 (3)	0.004 (2)	0.006 (3)	−0.005 (2)
C9	0.020 (3)	0.021 (3)	0.022 (3)	0.001 (2)	−0.001 (2)	−0.001 (2)
C10	0.019 (3)	0.034 (3)	0.037 (3)	−0.003 (3)	0.007 (3)	0.003 (3)
C11	0.021 (3)	0.032 (3)	0.044 (4)	0.010 (3)	0.001 (3)	0.003 (3)
C12	0.022 (3)	0.022 (3)	0.025 (3)	0.004 (2)	0.006 (2)	−0.003 (2)
C13	0.034 (3)	0.034 (3)	0.032 (3)	−0.001 (3)	0.009 (3)	−0.006 (3)
C14	0.031 (3)	0.030 (3)	0.038 (3)	−0.008 (3)	0.009 (3)	−0.008 (3)
C15	0.020 (3)	0.013 (2)	0.033 (3)	−0.001 (2)	0.004 (2)	0.002 (2)
C16	0.031 (3)	0.033 (3)	0.023 (3)	−0.003 (3)	0.001 (2)	0.000 (2)
C17	0.022 (3)	0.036 (3)	0.039 (3)	−0.007 (3)	0.000 (3)	0.003 (3)
C18	0.024 (3)	0.021 (3)	0.024 (3)	−0.006 (2)	0.000 (2)	−0.002 (2)
C19	0.018 (3)	0.030 (3)	0.038 (3)	−0.004 (2)	0.002 (3)	0.003 (2)
C20	0.021 (3)	0.028 (3)	0.022 (3)	0.002 (2)	0.004 (2)	0.001 (2)
C21	0.023 (3)	0.028 (3)	0.048 (4)	0.004 (3)	0.015 (3)	−0.002 (3)
C22	0.021 (3)	0.024 (3)	0.032 (3)	−0.004 (2)	0.003 (2)	0.001 (2)
C23	0.017 (3)	0.029 (3)	0.015 (2)	−0.003 (2)	0.005 (2)	0.002 (2)
C24	0.018 (3)	0.028 (3)	0.025 (3)	−0.001 (2)	0.007 (2)	0.003 (2)

### Geometric parameters (Å, °)

**Table:**

Gd1—O1	2.357 (4)	N4—C19	1.354 (8)
Gd1—O2^i^	2.465 (4)	N4—Ag1^iii^	2.147 (5)
Gd1—O3	2.383 (4)	C1—C2	1.388 (8)
Gd1—O5	2.393 (4)	C1—H1A	0.9300
Gd1—O7	2.399 (4)	C2—C3	1.383 (8)
Gd1—O8^i^	2.386 (4)	C2—H2A	0.9300
Gd1—O9	2.454 (4)	C3—C4	1.395 (7)
Gd1—O10	2.533 (4)	C3—C6	1.494 (7)
Ag1—N1	2.144 (5)	C4—C5	1.377 (8)
Ag1—N4^ii^	2.147 (5)	C4—H4A	0.9300
Ag2—N2^iii^	2.189 (5)	C5—H5A	0.9300
Ag2—N3	2.199 (5)	C7—C8	1.385 (9)
O1—C6	1.252 (7)	C7—H7A	0.9300
O2—C6	1.238 (7)	C8—C9	1.386 (8)
O2—Gd1^iv^	2.465 (4)	C8—H8A	0.9300
O3—C12	1.261 (7)	C9—C10	1.378 (8)
O4—C12	1.227 (7)	C9—C12	1.530 (8)
O5—C18	1.229 (7)	C10—C11	1.374 (8)
O6—C18	1.238 (7)	C10—H10B	0.9300
O7—C24	1.253 (7)	C11—H11A	0.9300
O8—C24	1.251 (7)	C13—C14	1.367 (9)
O8—Gd1^iv^	2.386 (4)	C13—H13A	0.9300
O9—H9A	0.8500	C14—C15	1.387 (8)
O9—H9B	0.8500	C14—H14A	0.9300
O10—H10C	0.8500	C15—C16	1.371 (8)
O10—H10A	0.8500	C15—C18	1.531 (8)
O11—N5	1.229 (9)	C16—C17	1.370 (9)
O12—N5	1.208 (9)	C16—H16A	0.9300
O13—N5	1.244 (10)	C17—H17A	0.9300
O14—H14C	0.8500	C19—C20	1.377 (8)
O14—H14B	0.8500	C19—H19A	0.9300
N1—C1	1.345 (8)	C20—C23	1.377 (8)
N1—C5	1.348 (8)	C20—H20A	0.9300
N2—C7	1.331 (8)	C21—C22	1.359 (9)
N2—C11	1.348 (8)	C21—H21A	0.9300
N2—Ag2^ii^	2.189 (5)	C22—C23	1.384 (8)
N3—C17	1.338 (8)	C22—H22A	0.9300
N3—C13	1.359 (8)	C23—C24	1.512 (7)
N4—C21	1.354 (8)		
			
O1—Gd1—O3	80.95 (15)	C2—C3—C6	121.5 (5)
O1—Gd1—O8^i^	117.84 (16)	C4—C3—C6	120.4 (5)
O3—Gd1—O8^i^	140.74 (14)	C5—C4—C3	120.1 (5)
O1—Gd1—O5	144.27 (17)	C5—C4—H4A	120.0
O3—Gd1—O5	101.65 (17)	C3—C4—H4A	120.0
O8^i^—Gd1—O5	82.71 (15)	N1—C5—C4	121.9 (6)
O1—Gd1—O7	77.42 (16)	N1—C5—H5A	119.1
O3—Gd1—O7	145.32 (15)	C4—C5—H5A	119.1
O8^i^—Gd1—O7	73.87 (15)	O2—C6—O1	125.6 (5)
O5—Gd1—O7	81.52 (18)	O2—C6—C3	118.8 (5)
O1—Gd1—O9	76.97 (17)	O1—C6—C3	115.4 (5)
O3—Gd1—O9	73.28 (16)	N2—C7—C8	123.3 (6)
O8^i^—Gd1—O9	141.49 (16)	N2—C7—H7A	118.4
O5—Gd1—O9	69.98 (15)	C8—C7—H7A	118.4
O7—Gd1—O9	75.62 (18)	C9—C8—C7	118.6 (6)
O1—Gd1—O2^i^	71.51 (16)	C9—C8—H8A	120.7
O3—Gd1—O2^i^	77.15 (14)	C7—C8—H8A	120.7
O8^i^—Gd1—O2^i^	77.31 (14)	C8—C9—C10	118.9 (5)
O5—Gd1—O2^i^	144.13 (14)	C8—C9—C12	120.6 (5)
O7—Gd1—O2^i^	119.97 (15)	C10—C9—C12	120.5 (5)
O9—Gd1—O2^i^	139.49 (14)	C11—C10—C9	118.6 (6)
O1—Gd1—O10	141.58 (15)	C11—C10—H10B	120.7
O3—Gd1—O10	71.24 (14)	C9—C10—H10B	120.7
O8^i^—Gd1—O10	74.21 (14)	N2—C11—C10	123.5 (6)
O5—Gd1—O10	69.25 (15)	N2—C11—H11A	118.2
O7—Gd1—O10	138.84 (14)	C10—C11—H11A	118.2
O9—Gd1—O10	117.66 (16)	O4—C12—O3	127.1 (5)
O2^i^—Gd1—O10	76.74 (14)	O4—C12—C9	117.6 (5)
N4^ii^—Ag1—N1	178.6 (2)	O3—C12—C9	115.3 (5)
N2^iii^—Ag2—N3	147.1 (2)	N3—C13—C14	123.0 (6)
C6—O1—Gd1	162.4 (4)	N3—C13—H13A	118.5
C6—O2—Gd1^iv^	132.5 (3)	C14—C13—H13A	118.5
C12—O3—Gd1	138.3 (4)	C13—C14—C15	119.3 (6)
C18—O5—Gd1	146.4 (4)	C13—C14—H14A	120.3
C24—O7—Gd1	161.8 (4)	C15—C14—H14A	120.3
C24—O8—Gd1^iv^	137.2 (4)	C16—C15—C14	118.0 (5)
Gd1—O9—H9A	99.5	C16—C15—C18	122.1 (5)
Gd1—O9—H9B	127.5	C14—C15—C18	119.8 (5)
H9A—O9—H9B	127.5	C17—C16—C15	119.6 (5)
Gd1—O10—H10C	104.3	C17—C16—H16A	120.2
Gd1—O10—H10A	121.4	C15—C16—H16A	120.2
H10C—O10—H10A	104.5	N3—C17—C16	123.6 (6)
H14C—O14—H14B	129.5	N3—C17—H17A	118.2
C1—N1—C5	118.0 (5)	C16—C17—H17A	118.2
C1—N1—Ag1	121.4 (4)	O5—C18—O6	127.3 (6)
C5—N1—Ag1	120.6 (4)	O5—C18—C15	116.5 (5)
C7—N2—C11	117.1 (5)	O6—C18—C15	116.2 (5)
C7—N2—Ag2^ii^	121.6 (4)	N4—C19—C20	122.8 (5)
C11—N2—Ag2^ii^	121.0 (4)	N4—C19—H19A	118.6
C17—N3—C13	116.4 (5)	C20—C19—H19A	118.6
C17—N3—Ag2	121.3 (4)	C23—C20—C19	118.9 (5)
C13—N3—Ag2	122.2 (4)	C23—C20—H20A	120.5
C21—N4—C19	116.7 (5)	C19—C20—H20A	120.5
C21—N4—Ag1^iii^	122.2 (4)	N4—C21—C22	123.4 (6)
C19—N4—Ag1^iii^	121.0 (4)	N4—C21—H21A	118.3
O12—N5—O11	121.1 (9)	C22—C21—H21A	118.3
O12—N5—O13	120.3 (7)	C21—C22—C23	119.0 (5)
O11—N5—O13	118.4 (8)	C21—C22—H22A	120.5
N1—C1—C2	123.3 (6)	C23—C22—H22A	120.5
N1—C1—H1A	118.4	C20—C23—C22	118.9 (5)
C2—C1—H1A	118.4	C20—C23—C24	120.2 (5)
C3—C2—C1	118.6 (5)	C22—C23—C24	120.8 (5)
C3—C2—H2A	120.7	O7—C24—O8	125.9 (5)
C1—C2—H2A	120.7	O7—C24—C23	116.8 (5)
C2—C3—C4	118.1 (5)	O8—C24—C23	117.2 (5)

Symmetry codes: (i) *x*, −*y*+1/2, *z*+1/2; (ii) *x*+1, *y*, *z*; (iii) *x*−1, *y*, *z*; (iv) *x*, −*y*+1/2, *z*−1/2.

### Hydrogen-bond geometry (Å, °)

**Table:**

*D*—H···*A*	*D*—H	H···*A*	*D*···*A*	*D*—H···*A*
O9—H9A···O6	0.85	2.13	2.771 (7)	132
O9—H9B···O2^iv^	0.85	2.03	2.811 (6)	153
O10—H10A···O6^v^	0.85	2.12	2.954 (6)	165
O10—H10C···O4	0.85	1.84	2.662 (6)	162
O14—H14B···O12^vi^	0.85	2.27	2.960 (9)	139
O14—H14C···O13^vii^	0.85	2.01	2.779 (10)	151

Symmetry codes:  (iv) *x*, −*y*+1/2, *z*−1/2; (v) *x*, *y*, *z*+1; (vi) *x*+1, −*y*+1/2, *z*−1/2; (vii) *x*+1, −*y*+1/2, *z*+1/2.
